# AICAR induces S phase cell cycle arrest in ESCC by targeting the AMPK/mTORC1 and mTORC2/AKT pathway

**DOI:** 10.3389/fonc.2026.1975805

**Published:** 2026-10-22

**Authors:** Muping Zou, Tianrui Zhang, Lingbin He, Jianhong Li, Ze Lin, Chenxi Jia, Kaixuan Ma, Lincui Wang, Shuya Xu, Bozhi Cai, Libiao Wu, Chunhong Ren, Thomas Ming Swi Chang, Pingnan Sun, Xin Zhang

**Affiliations:** 1 Laboratory of Molecular Cardiology, The First Affiliated Hospital of Shantou University Medical College, Shantou, Guangdong, China; 2 Shantou University Medical College, Shantou, Guangdong, China; 3 Clinical research center, The First Affiliated Hospital of Shantou University Medical College, Shantou, Guangdong, China; 4 International Medical Service Center, The First Affiliated hospital of Shantou University Medical College, Shantou, Guangdong, China; 5 Artificial Cells and Organs Research Centre, Departments of Physiology, Medicine and Biomedical Engineering, Faculty of Medicine & Biomedical Sciences, McGill University, Montreal, QC, Canada; 6 Department of Gynecology of the First Affiliated Hospital, Shantou University Medical College, Shantou, Guangdong, China; 7 Department of Stem Cell Research Center, Shantou University Medical College, Shantou, Guangdong, China; 8 Engineering Research Center of key technique for biotherapy of Guangdong Province, Shantou, Guangdong, China

**Keywords:** AICAR, AMPK, cell cycle arrest, chemo sensitization, ESCC

## Abstract

**Background:**

5-Aminoimidazole-4-carboxamide-1-β-4-ribofuranoside (AICAR), an AMP-activated protein kinase (AMPK) agonist, exhibits potent antitumor capacity across various malignancies. However, its efficacy and underlying mechanisms in esophageal squamous cell carcinoma (ESCC) remain largely unexplored.

**Methods:**

We treated HEEC, TE1, and TE13 cells with AICAR and assessed proliferation, cell cycle distribution, and expression of key signaling proteins, followed by SN38 cotreatment to evaluate apoptosis and chemosensitization.

**Results:**

AICAR treatment suppressed proliferation in all three cell lines but induced a distinct S-phase arrest specifically in ESCC cells. Mechanistically, AICAR activated AMPK, which concurrently inhibited the mTORC1 pathway via TSC2 phosphorylation and activated the mTORC2/AKT pathway through FOXO3-mediated transcriptional upregulation of Rictor. A critical difference was observed between cell types: in HEEC cells, AICAR treatment upregulated the tumor suppressor PTEN, which prevented full AKT activation. In contrast, ESCC cells, with unaltered PTEN and dysfunctional p53, sustained AKT hyperactivation. This drove cells into S-phase, where they were subsequently arrested due to the parallel inhibition of mTORC1. Furthermore, synchronizing ESCC cells in the S-phase with AICAR, followed by its withdrawal and subsequent treatment with SN38, significantly enhanced apoptosis.

**Conclusion:**

Our findings reveal that AICAR induces S-phase arrest in ESCC cells by dually modulating the mTORC1 and mTORC2/AKT signaling pathways. This differential effect, stemmed from dysfunctional p53/PTEN axis in ESCC cells, distinguishes ESCC cells from normal epithelial cells. Together, these results suggest that AICAR synchronizes ESCC cells in the S phase, thereby sensitizing them to S-phase-specific agents like SN38 and representing a potential novel strategy for ESCC treatment.

## 1 Introduction

Esophageal cancer ranks as the sixth leading cause of cancer related mortality worldwide (1). Patients with early-stage esophageal cancer usually remain asymptomatic or experience only subtle, non-specific symptoms, resulting in diagnosis at advanced stages in a significant proportion of cases (2). Marked by dismal prognosis and a scarcity of effective therapeutic alternatives, esophageal cancer yields a 5-year survival rate of less than 20% (3). Chemotherapy remains the primary therapeutic modality for advanced esophageal malignancy. However, the development of tumor chemoresistance constitutes a critical bottleneck restricting therapeutic efficacy (4). Therefore, there is an urgent need to develop strategies employing chemical sensitizers to overcome drug resistance, augment the chemosensitivity of tumor cells, and ultimately improve clinical survival outcomes.

AMPK is a highly conserved serine/threonine protein kinase activated under metabolic stress conditions such as ischemia, hypoxia, and glucose deprivation (5–7). Upon activation, AMPK phosphorylates downstream targets such as TSC2 (8) and FOXO3a (9), thereby inhibiting anabolic pathways, stimulating catabolic processes, and sustaining cellular energy homeostasis. Accumulating evidence has demonstrated over the past decade that AMPK not only regulates cellular metabolism but also participates in various biological processes including cell cycle progression, apoptosis, autophagy, invasion, and metastasis (10–12). Notably, AMPK exerts context-dependent dual roles in oncogenesis: it predominantly functions as a tumor suppressor at the early stages of tumorigenesis (13), yet sustained AMPK activation during established tumor progression enables malignant cells to adapt to adverse microenvironmental stress, further driving the acquisition of chemoresistance (14, 15). This paradoxical dual functionality renders AMPK an attractive yet challenging therapeutic target for anti-cancer interventions.

AICAR, a well-characterized pharmacological activator of AMPK (16, 17), has been shown to suppresses the malignant phenotype of various tumor cell types both *in vivo* and *in vitro* through mechanisms, such as the inhibition of cell proliferation (18), induction of cell cycle arrest (19), promotion of apoptosis (20), and generation of intracellular reactive oxygen species (21). These antitumor effects have been documented in pancreatic cancer (22), multiple myelomas (23), breast cancer (24), melanoma (25), prostate cancer (26), childhood acute lymphoblastic leukemia (27) and cervical cancer (28). Notably, AICAR exhibits minimal cytotoxicity toward non-tumorigenic cells, suggesting its tumor-preferential cytotoxicity (29, 30). Despite these promising preclinical results, the therapeutic potential of AICAR in ESCC remains largely unexplored. Furthermore, it remains unknown whether AICAR exerts distinct biological impacts on normal versus malignant esophageal cells, as well as the molecular mechanisms underlying such potential discrepancies.

In this study, we reveal that AICAR induces distinct cell cycle responses in normal esophageal epithelial cells versus ESCC cells. Mechanistically, AMPK activated by AICAR concomitantly inhibits mTORC1 signaling while simultaneously activating the mTORC2/AKT pathway. Crucially the differential expression of PTEN between normal and malignant esophageal cells influences AKT activation, thereby determining cell cycle fates. In addition, AICAR pretreatment sensitizes ESCC cells to S-phase-specific agents, such as SN38 (7-Ethyl-10-hydroxycamptothecin, the active metabolite of irinotecan) and 5-fluorouracil(5-Fu). Collectively, these findings highlight the therapeutic potential of combining AICAR with conventional chemotherapy as a promising therapeutic strategy against ESCC.

## 2 Materials and methods

### 2.1 Materials

Reagents were purchased from the indicated vendors. Antibodies against AMPK (#2795), FOXO3(#2497), p-AMPK(Thr172) (#2531), p-FOXO3(Ser413) (#8174), p-FOXO3(Ser253) (#2497), AKT (#9272), p-AKT(Ser473) (#4060), p-AKT(Thr308) (#9275), Rb (#9313), p-Rb (#9307), Rictor (#2140), cl-Caspase-3(#9661), p70S6K(#9202), p-p70S6K(Thr389)(# 9205),S6(#2217), p-S6 (Ser235/236) (#2211) and GAPDH (#2118) were obtained from Cell Signaling. Antibodies against p21^Cip1^(sc-469), p27^Kip1^(sc-393380), p53(sc-6243) and PTEN (sc-7974) were supplied by Santa Cruz Biotechnology. TSC2(24601-1-AP) and p-TSC2 (Ser1387) (80553-2-RR) were purchased from Proteintech. AICAR (2627-69-2) and SN38(HY-13704) were obtained from Selleck. 5-Fu (03738) was obtained from Sigma.

### 2.2 Cell culture

Human esophageal epithelial cells HEEC cells were purchased from ScienCell Research Laboratories (Carlsbad, CA, Cat#2720). Human esophageal squamous cell carcinoma cell lines TE1(BNCC100151) and TE13(BNCC100437) were purchased from Beina Biological Technology Co., Ltd. (Bejing, China). All three cell lines were cultured in RPMI 1640 medium, supplemented with 10% of fetal bovine serum (FBS, Gibco) and maintained at 37 °C in a humidified incubator supplied with 5% CO_2_.

### 2.3 CCK8

TE1, TE13, and HEEC Cells were seeded into 96-well plates at a density of 4 × 10³ per well and incubated overnight. For working concentration screening, cells were treated with 0, 250, 500, and 1000 μM AICAR for 48 h. Cell viability was detected using CCK8 reagent (Dojindo, Japan), and the rationale for optimal dose selection is shown in **Supplementary Figure S1**. For time-dependent proliferation analysis, cells were exposed to 500 μM AICAR for 24, 48, and 72 hours. At each indicated time point, the culture medium was replaced with fresh medium containing 10% CCK8 reagent (Dojindo, Japan). After 1 h incubation at 37 °C, the absorbance value of each well was measured at 450 nm using a microplate spectrophotometer.

### 2.4 Ki67 Immunostaining

Cells were collected at 0, 24, 48, and 72 hours following AICAR treatment. For sequential chemosensitization assays, cells were pre-incubated with AICAR for 48 hours, followed by additional 24 or 48 hours of culture in AICAR-free medium. Briefly, harvested cells were washed twice with precooled phosphate-buffered saline (PBS), fixed with 4% paraformaldehyde for 15 minutes, permeabilized with 0.1% Triton X-100 for 15 minutes, and blocked with 10% goat serum for 30 minutes at room temperature. Subsequently, cells were then incubated with Ki67 primary antibody working solution for 10 minutes, and the fluorescence intensity was quantified using an Accuri C6 flow cytometer.

### 2.5 Annexin V—PI staining for apoptotic cells

Cells were seeded in 6-well plates and incubated overnight. Cells were pretreated with AICAR (500 μM) for 48 hours, followed by SN38 (40 nM) treatment for an additional 48 hours; untreated cells were set as the blank controls. Cells were harvested by trypsinization, washed twice with PBS, and resuspended at a concentration of 1 ×10^5^ cells/mL. Apoptosis was assessed using an Annexin V-FITC/PI Apoptosis Detection Kit (C1062L, Beyotime, China) following the manufacturer’s instructions. Stained samples were analyzed on a BD Accuri C6 flow cytometer (BD Biosciences, USA). Viable cells exhibited double-negative for both annexin V and with propidium iodide (PI). Early apoptotic cells were Annexin V-positive yet PI-negative, while late apoptotic/necrotic cells were positive for both annexin V and PI.

### 2.6 Cell cycle analysis

Following AICAR treatment, TE1, TE13, and HEEC cells were collected at 0, 24, 48, and 72 hours. Cells were washed twice with precooled PBS and fixed in 95% ethanol overnight at 4 °C. Fixed cells were stained with PI using a Cell Cycle and Apoptosis Detection Kit (C1052, Beyotime, China) according to the manufacturer’s protocol. Cell cycle distribution was determined using an Accuri C6 flow cytometer.

### 2.7 Western blotting

Cells were harvested at 0, 24, 48, and 72 hours after AICAR treatment, washed twice with precooled PBS, and then lysed in ice-cold RIPA buffer for 30 minutes. Lysates were clarified by centrifugation at 12,000 g for 15 minutes at 4 °C. Equal amounts of total protein were separated by 10% SDS-PAGE and subsequently electrotransferred onto PVDF membranes. The membranes were blocked with 5% non-fat milk in TBST for 1 hour at room temperature, then incubated with primary antibodies overnight at 4 °C. After three sequential 10 minutes washes in TBST, membranes were incubated with HRP-conjugated secondary antibodies for 1 hours at room temperature. After another round of TBST washes, protein signals were visualized using an enhanced chemiluminescence detection system (Bio-Rad ChemiDoc XRS+, USA). Band intensities were quantified using ImageJ. For non-phosphorylated proteins, signals were normalized to the loading control. For phosphorylated proteins, the gray value of the phosphorylated protein was first normalized to its corresponding loading control, and the gray value of the total protein was separately normalized to the loading control. The final phosphorylation level was calculated as the ratio of (p-protein/loading control) to (total-protein/loading control), and then normalized to the control group (set as 1.0). Statistical analyses were performed as described in the Statistical Analysis section.

### 2.8 Statistical analysis

All quantitative data are presented as mean ± standard error of the mean (SEM). For multiple group comparisons, one-way analysis of variance (ANOVA) was performed. Dunnett’s *post-hoc* test was used to compare each treatment time point with the 0 h control. Tukey’s *post-hoc* test was used for pairwise comparisons among treatment groups in the combination therapy experiments. A *p*-value of less than 0.05 was considered statistically significant. All statistical analyses were conducted using GraphPad Prism 10.1 software.

## 3 Results

### 3.1 AICAR inhibits the proliferation of ESCC cells

To evaluate the impact of AICAR on cell proliferation, ESCC cell lines (TE1 and TE13), and normal esophageal epithelial cells (HEEC) were treated with 500 μM AICAR for 0, 24, 48, and 72 hours. CCK8 assay showed that AICAR significantly suppressed the proliferative capacity of all three cell lines in a time-dependent manner compared with the untreated controls (**Figure 1A**). Consistent with these results, flow cytometric analysis of Ki67-positive cells corroborated a marked suppression of proliferation across all three lines following AICAR treatment (**Figure 1B**).

**Figure 1:**
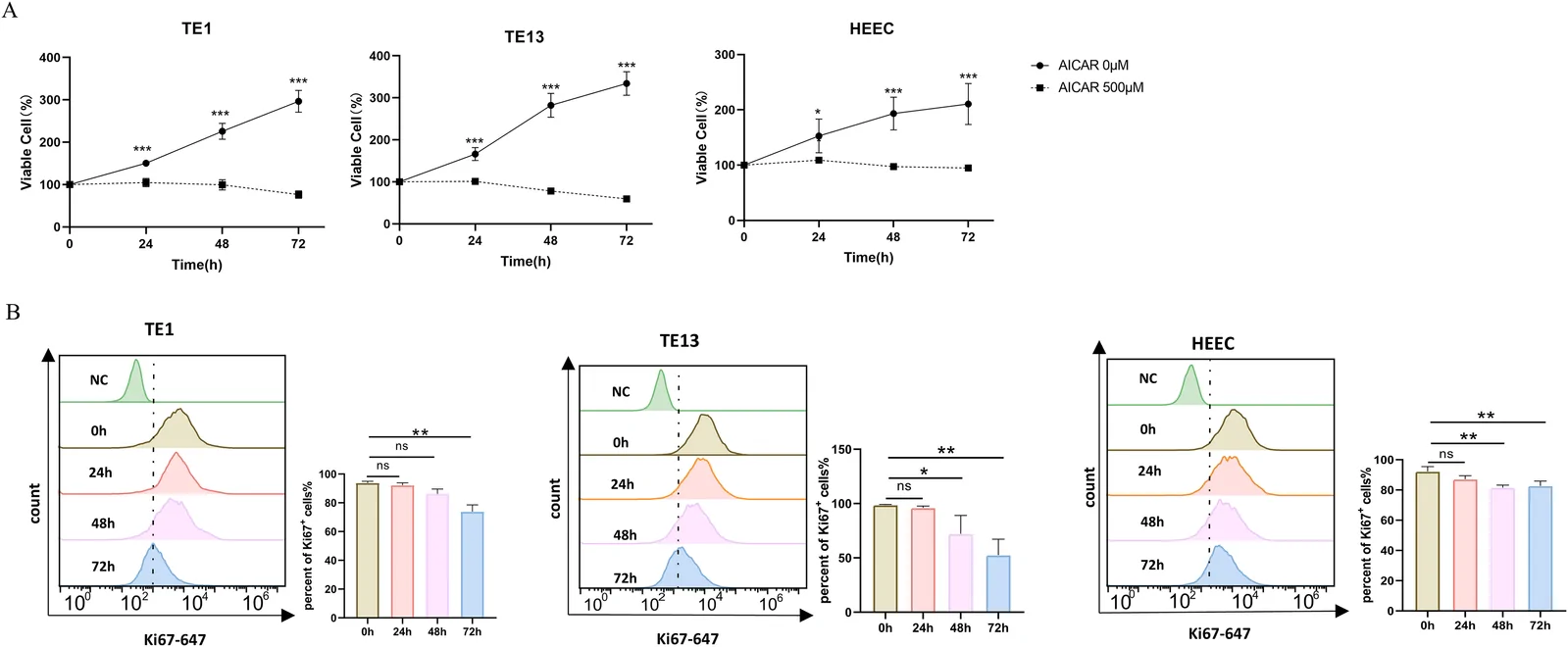
AICAR inhibits proliferation of TE1, TE13 and HEEC cells. **(A)** Cell viability was measured by CCK-8 assay at 0, 24, 48 and 72 hours after treatment with 500 μM AICAR. The viability of control cells at 0 h was set to 100%. **(B)** Ki67-positive cells were quantified by flow cytometry at the indicated time points. Data are presented as mean ± SEM (n = 3). *p < 0.05, **p < 0.01, ***p < 0.001 vs. untreated controls.

### 3.2 AICAR induces S phase arrest in ESCC cells

Cell cycle distribution was assessed by PI staining following AICAR treatment. Notably, AICAR induced markedly distinct cell cycle responses between malignant and normal cells. TE1 and TE13 cells exhibited time-dependent S phase arrest after AICAR treatment. Specifically, the S phase fraction in TE1 cells increased from 33.85% ± 3.52% in untreated controls (0 hour) to 52.87% ± 2.44% after 72 hours of AICAR treatment (*p* < 0.05, **Figure 2A**). A more pronounced accumulation was observed in TE13 cells, in which the S-phase fraction increased from 47.42% ± 2.25% to 73.83% ± 9.07% (*p* < 0.05, **Figure 2A**). In contrast, HEEC cells showed no significant cell cycle alterations, with the S-phase fraction remaining stable throughout the treatment period (37.85% ± 3.03% untreated vs. 42.79% ± 5.25% at 72 hours, *p* > 0.05). Although a marginal, transient increase in the G0/G1 population was detected at 24 and 48 hours in HEEC cells, this shift returned to baseline levles by 72 hours. Collectively, these data demonstrate that AICAR induces S-phase arrest with selectivity for ESCC cells over normal esophageal epithelial cells.

**Figure 2:**
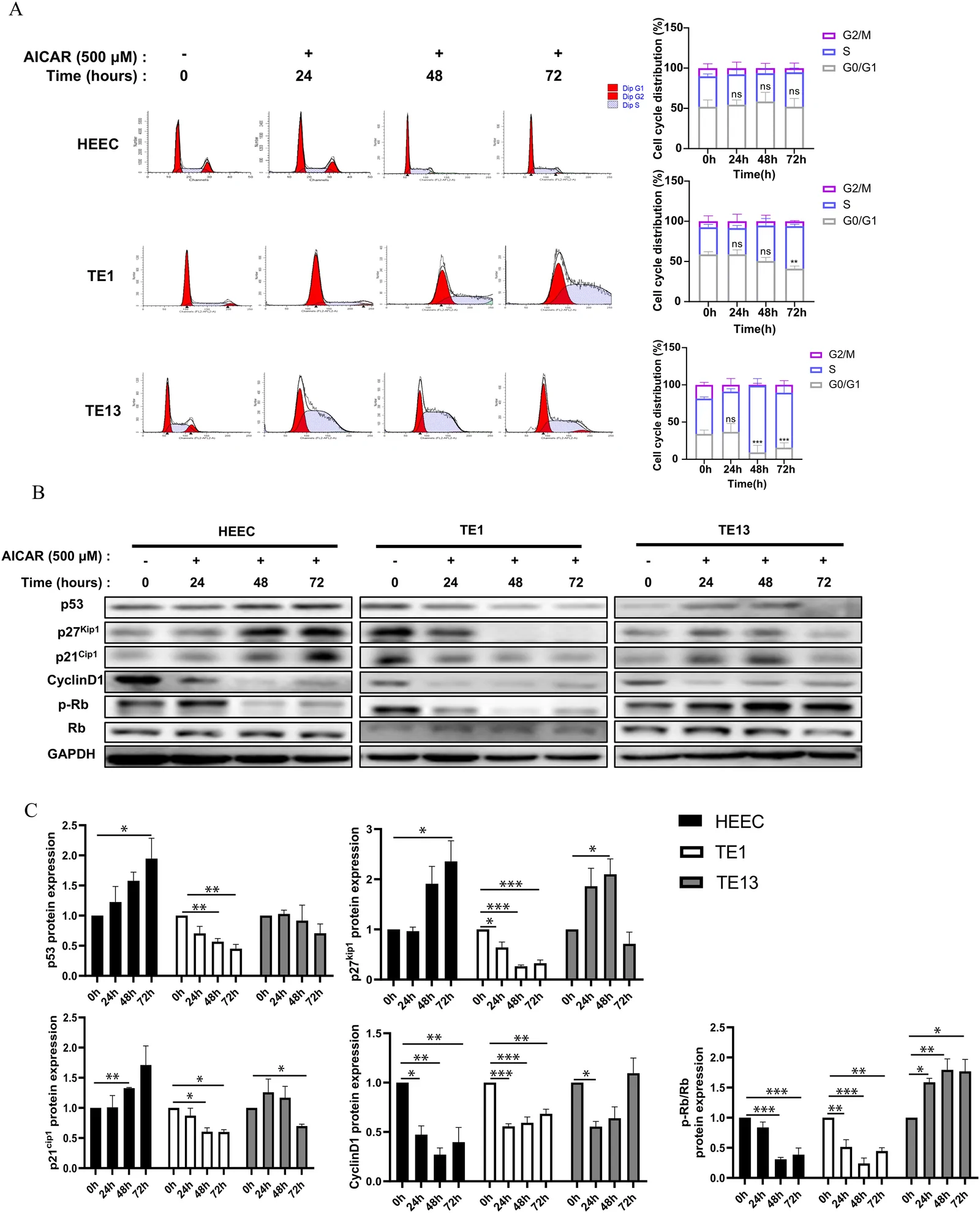
AICAR induces S-phase arrest selectively in ESCC cells. **(A)** Cell cycle distribution was analyzed by flow cytometry following PI staining in TE1, TE13 and HEEC cells treated with AICAR for 0, 24, 48 or 72 hours. Data represent mean ± SEM, n=3, * *p* < 0.05. **(B)** The protein levels of p21^Cip1^, p27^Kip1^ and p53, CyclinD1 and p-Rb after AICAR treatment at 0,24,48 and 72 h were detected by Western blot. **(C)** Quantitative densitometric analysis of p21^Cip1^, p27^Kip1^ and p53, CyclinD1 and p-Rb. Data represent mean ± SEM from three independent experiments. *p < 0.05, **p < 0.01, ***p < 0.001 versus control group. No significant difference was observed at other time points compared with 0 h.

### 3.3 AICAR differentially regulates cell cycle-related proteins in ESCC and normal cells

To elucidate the mechanisms underlying divergent cell cycle responses, we examined the abundance of key cell cycle regulators by western blotting. In HEEC cells, the protein levels of p21^Cip1^, p27^Kip1^ and p53 gradually increased with prolonged AICAR treatment (**Figures 2B, C**). Conversely, Cyclin D1 expression and phosphorylated retinoblastoma (p-Rb) protein levels declined at 24 hours and 48 hours but partially recovered at 72 hours upon AICAR treatment. In TE1 cells, prolonged AICAR treatment gradually reduced the protein levels of p21^Cip1^, p27^Kip1^ and p53. By contrast, Cyclin D1 and p-Rb were initially reduced and then partially restored, mirroring the kinetics observed in HEEC cells. For TE13 cells, a transient upregulation of p21^Cip1^, p27^Kip1^ and p53 was detected at 24 hours post-treatment before declining, accompanied by sustained Rb hyperphosphorylation. Collectively, the progressive downregulation of p21^Cip1^, p27^Kip1^ and p53, coupled with elevated p-Rb levels in TE1 and TE13 cells, compromised the G1/S checkpoint and facilitated S-phase entry.

### 3.4 AICAR activates AMPK and suppresses mTORC1 signaling

Previous studies have established AICAR as a selective pharmacological activator of AMPK (17). Western blot analysis confirmed that AICAR treatment increased phosphorylated AMPK (p-AMPK) levels in a time-dependent manner across all three cell lines, while total AMPK levels remained unchanged (**Figures 3A, B**). To evaluate mTORC1 activity, downstream of AMPK, we monitored the phosphorylation status of TSC2, S6, and 70S6K. AICAR treatment elevated p-TSC2 levels and reduced p-70S6K and p-S6 levels in all three cell lines (**Figures 3C, D**), indicating a robust suppression of mTORC1 signaling. This molecular event likely accounts for the observed inhibition of cell proliferation. However, since mTORC1 inhibition alone failed to explain the divergent cell cycle phenotypes among the three cell lines, further investigation into additional signaling pathways was warranted.

**Figure 3:**
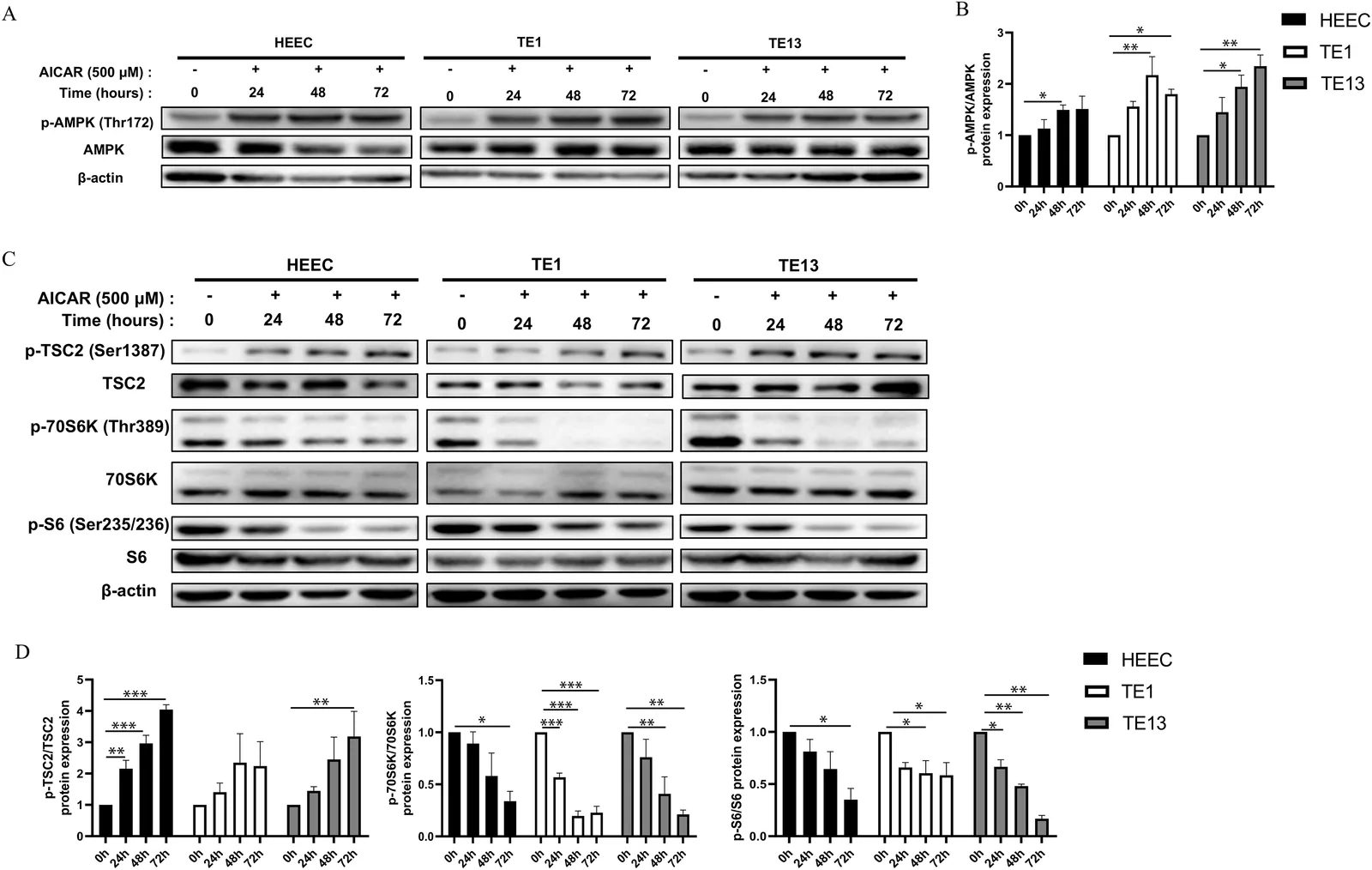
AICAR activates AMPK and suppresses mTORC1 signaling. **(A)** Western blot analysis of phosphorylated AMPK (Thr172) and total AMPK after AICAR treatment for 0, 24, 48 or 72 hours. **(B)** Quantitative densitometric analysis of p-AMPK. Data represent mean ± SEM from three independent experiments. *p < 0.05, **p < 0.01 versus control group. No significant difference was observed at other time points compared with 0 h. **(C)** Western blot analysis of phosphorylated TSC2 (Ser1387), 70S6K (Thr389) and S6 (Ser235/236) in cells treated with AICAR for 0, 24, 48 or 72 hours. **(D)** Quantitative densitometric analysis of p-TSC2 (Ser1387), p-70S6K (Thr389) and p-S6 (Ser235/236). Data represent mean ± SEM from three independent experiments. *p < 0.05, **p < 0.01, ***p < 0.001 versus control group. No significant difference was observed at other time points compared with 0 h.

### 3.5 AICAR activates the mTORC2/AKT pathway via FOXO3a-mediated Rictor upregulation

Phosphorylation of FOXO3a at Ser413 by AMPK enhances its activity (9). As a transcription factor, FOXO3a directly targets the Rictor promoter to activate its expression (31). Given that Rictor is a core component of mTORC2, its upregulation leads to mTORC2 activation, and AKT phosphorylation at Ser473 (31, 32). Consistent with this mechanism, Western blot analysis revealed that p-FOXO3a (Ser413), Rictor and p-AKT (Ser473) levels progressively increased in all three cell lines following AICAR treatment (**Figure 4**), indicating sustained mTORC2 activation.

**Figure 4:**
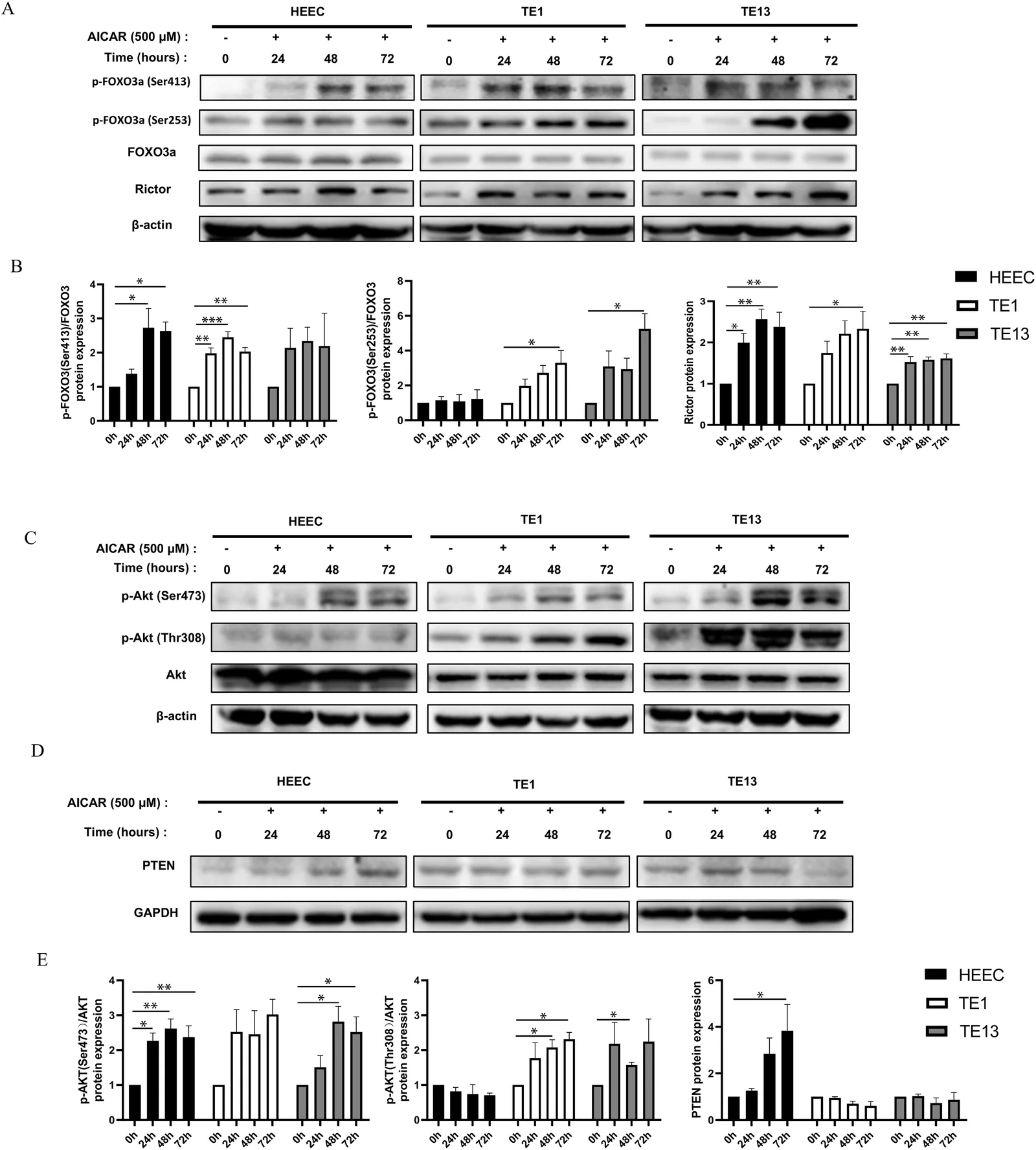
AICAR activated mTORC2/AKT pathway, with differential AKT activation determined by PTEN expression. **(A)** Western blot analysis of Rictor expression and FOXO3a phosphorylation at Ser413 and Ser253. **(B)** Quantitative densitometric analysis of Rictor, p-Foxo3 (Ser413) and p-Foxo3 (Ser253). Data represent mean ± SEM from three independent experiments. *p < 0.05, **p < 0.01, ***p < 0.001 versus control group. No significant difference was observed at other time points compared with 0 h. **(C)** Western blot analysis of AKT phosphorylation at Ser473 and Thr308. **(D)** Western blot analysis of PTEN expression. **(E)** Quantitative densitometric analysis of p-AKT (Ser473), p-AKT (Thr308) and PTEN. Data represent mean ± SEM from three independent experiments. *p < 0.05, **p < 0.01, ***p < 0.001 versus control group. No significant difference was observed at other time points compared with 0 h. All proteins were examined in TE1, TE13 and HEEC cells after AICAR treatment for 0, 24, 48 or 72 hours.

### 3.6 Fully activated AKT may be related to the entry of cells into the S phase

Complete AKT activation requires dual phosphorylation at both Thr308 and Ser473 residues (33, 34). In TE1 and TE13 cells, AICAR treatment induced sustained elevation of p-AKT (Thr308), whereas no significant change was detected in HEEC cells (**Figures 4C, E**). Activated AKT suppresses the transcriptional activity of FOXO3a by phosphorylating it at Ser253, which facilitates FOXO3a nuclear export and subsequent degradation (35). AICAR treatment markedly increased FOXO3a phosphorylation at Ser253 in TE1 and TE13 cells, while HEEC cells showed no significant change (**Figures 4A, B**), consistent with cell-type-specific differences in p-AKT (Thr308) status. These data demonstrate that AKT is fully activated in ESCC cells, driving cell cycle progression into the S phase. However, concomitant mTORC1 inhibition blocks further cell cycle progression, resulting in S-phase arrest.

### 3.7 PTEN expression dictates differential AKT activation

PTEN, a negative regulator of PI3K/AKT signaling, suppresses AKT activity through dephosphorylation (36). Western blot analysis showed that PTEN expression progressively increased in HEEC cells following AICAR treatment. In contrast, PTEN levels remained largely unchanged or slightly decreased in TE1 and TE13 cells (**Figures 4D, E**). Such cell-type-specific regulation of PTEN accounts for the distinct AKT activation patterns: PTEN upregulation in HEEC cells restrains AKT activity, while the failure to upregulate PTEN in ESCC cells permits full AKT activation.

### 3.8 AICAR pretreatment enhances chemosensitivity via S-phase synchronization

Given that AICAR selectively arrests ESCC cells in S phase, we next investigated whether this synchronization could sensitize them to S-phase-specific chemotherapeutic agents. TE1 and TE13 cells were pretreated with AICAR for 48 hours, followed by drug withdrawal and subsequent treatment with SN38 (the active metabolite of irinotecan) for another 48 hours. Ki67 staining confirmed that cell proliferation recovered within 24 hours after AICAR withdrawal (**Figure 5A**). Annexin V/PI double staining revealed that the combined treatment group (AICAR pretreatment plus SN38) exhibited a markedly elevated apoptotic rate compared to either monotherapy group (**Figure 5B**). Consistently, Western blot analysis showed that cleaved caspase-3 levels were significantly elevated in the combination treatment group (**Figures 5C, D**). Furthermore, AICAR pretreatment also sensitized ESCC cells to 5-Fu, another S-phase-specific chemotherapeutic agent. Notably, the apoptotic rate in the AICAR + 5-Fu (1 μg/mL) group was comparable to that in the 5-Fu alone at a 5-fold higher concentration (5 μg/mL) group (albeit with no statistically significant difference), indicating that AICAR pretreatment substantially reduces the effective chemotherapeutic dose of 5-Fu (**Supplementary Figure 2**). Taken together, these results demonstrate that AICAR pretreatment enhances the chemosensitivity of ESCC cells to S-phase-specific chemotherapeutic agents.

**Figure 5:**
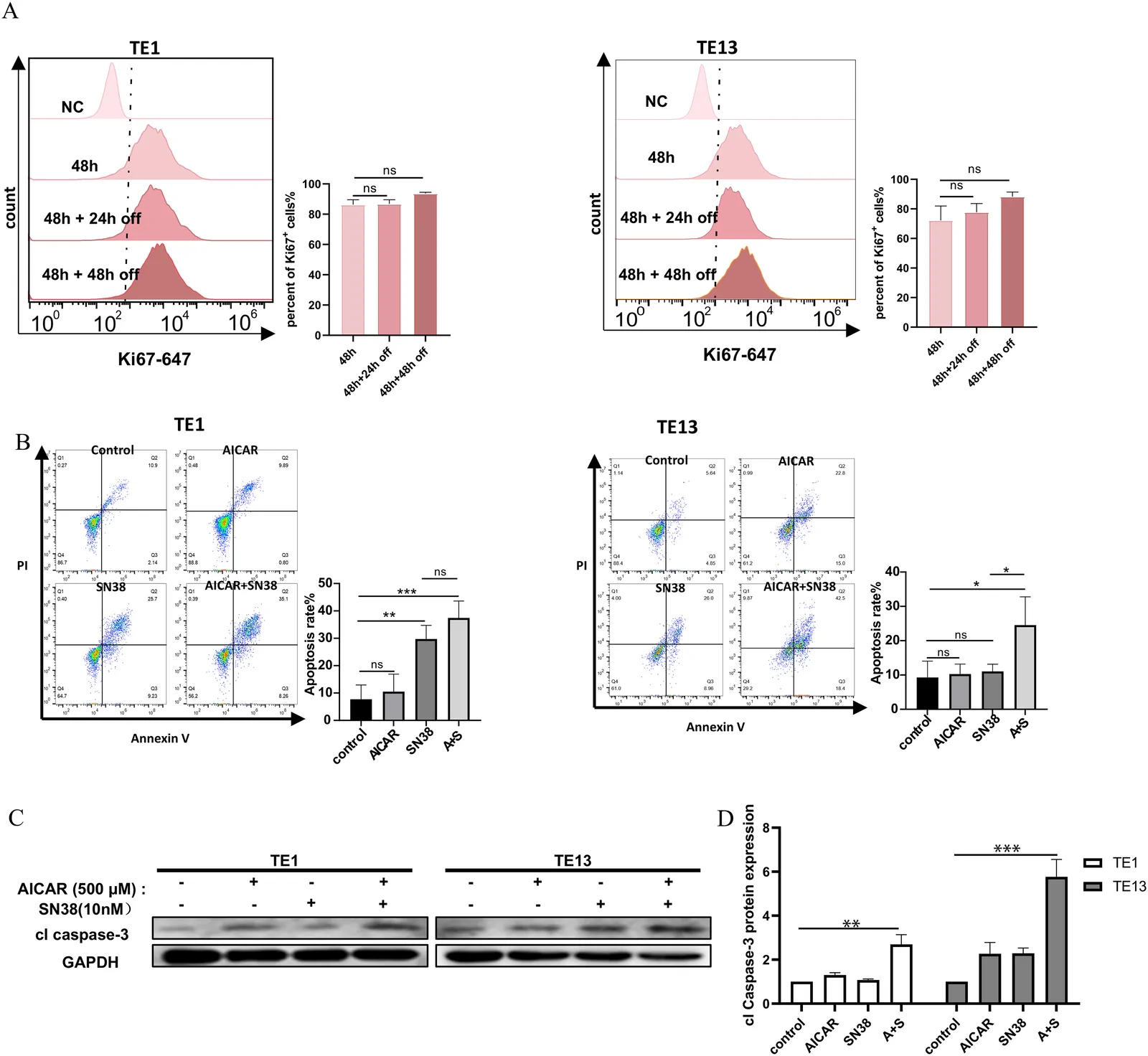
AICAR pretreatment enhances sensitivity of ESCC cells to SN38. **(A)** Ki67-positive cells were quantified by flow cytometry at 0, 24 and 48 hours after AICAR withdrawal. **(B)** Apoptosis was assessed by Annexin V/PI double staining in TE1 and TE13 cells pretreated with AICAR for 48 hours followed by SN38 treatment for 48 hours. **(C)** Western blot analysis of cleaved Caspase-3 expression in TE1 and TE13 cells subjected to the indicated treatments. **(D)** Quantitative densitometric analysis of cleaved Caspase-3. Data represent mean ± SEM from three independent experiments. *p < 0.05, **p < 0.01, ***p < 0.001 versus control group.

## 4 Discussion

AMPK serves as a critical cellular energy sensor that integrates nutrient availability with cell growth and proliferation. In the present study, we demonstrated that AICAR, a pharmacological AMPK activator, suppresses proliferation of both normal esophageal epithelial cells (HEEC) and ESCC cell lines (TE1 and TE13) through inhibition of mTORC1 pathway. However, AICAR induced divergent cell cycle responses: HEEC cells showed no significant cell cycle alteration, whereas ESCC cells were arrested in the S phase. This discrepancy likely arises from distinct PTEN/p53 regulatory status between normal and malignant cells, which may contribute to the differential extent of AKT activation. Furthermore, AICAR-mediated S-phase synchronization sensitizes ESCC cells to S-phase-specific chemotherapeutic agents such as including SN38 and 5-Fu.

The finding that AMPK activation universally suppresses proliferation across diverse cell types aligns with its well-recognized function as an energy sensor that restricts anabolic processes under metabolic stress (6, 17). Mechanistically, AICAR treatment triggers AMPK-mediated phosphorylation of TSC2, which subsequently inhibits mTORC1 signaling (8). This inhibition reduces the phosphorylation of 70S6K and S6, thereby suppressing ribosome biogenesis and protein translation (37–40). The consistent activation of this canonical pathway in all three cell lines provides a molecular rationale for the broad-spectrum antiproliferative efficacy of AICAR.

Although AMPK-mediated growth suppression occurs across various cell types, its cell cycle-modulating effects are highly cell-type dependent. In a broad spectrum of cancer cell types, including breast (41), hepatocellular (42), glioma (43) and renal cell carcinoma (44), AMPK activation commonly induces G0/G1 arrest through the p53/p21^Cip1^ axis (45), disruption of the Rb-E2F complex (46), and suppression of mTOR signaling (47). In our study, however, AICAR treatment induced prominent S phase arrest rather than G0/G1 phase arrest in ESCC cell, accompanied by a concomitant reduction in the G0/G1 population. By contrast, HEEC cells displayed only a mild and reversible G0/G1 phase delay. Such phenotypic discrepancies indicate that AMPK may modulate cell cycle progression in ESCC cells via a distinct regulatory pattern, which differs from the pathways previously described in other tumor types.

FOXO3a is a transcription factor that directly transactivates p21^Cip1^ and p27^Kip1^ while repressing cyclin D1 expression (48, 49). p21^Cip1^ and p27^Kip1^ suppress the activity of multiple cyclin-dependent kinases, arresting cells at G0/G1 phase. Conversely, cyclin D1 facilitates Rb phosphorylation and thereby drives progression into S-phase (50). In addition, p53 serves as an upstream regulator of p21^Cip1^ and p27^Kip1^ and engages in reciprocal feedback regulation with FOXO3a (51, 52). In HEEC cells harboring functional p53, AICAR treatment upregulated p53, p21^Cip1^ and p27^Kip1^, leading to transient suppression of cyclin D1 and p-Rb. This pattern reflects activation of the canonical G1/S checkpoint. However, as cells gradually adapt to metabolic stress, cyclin D1 and p-Rb levels partially recovered, allowing cells to maintain homeostatic cell cycle distribution. In contrast, TE1 and TE13 cells, which carry dysfunctional p53 (53), failed to upregulate p21^Cip1^ and p27^Kip1^ expression upon AICAR treatment. These alterations were associated with a reduced G0/G1 population and accumulation of cells within the S phase. Concurrent mTORC1 suppression impairs *de novo* nucleotide synthesis, which diminishes the dNTP pool required for DNA replication (54). The shortage of dNTPs blocks DNA elongation and consequently leads to S-phase arrest. Our observations align with a previous study by Theodoropoulou et al., which demonstrated that AICAR induces S-phase arrest in retinoblastoma cells through p21^Cip1^ downregulation (20). These data support a conserved mechanism whereby AICAR modulates cell cycle progression via p21^Cip1^ regulation.

A notable finding from this study is the concomitant activation of mTORC2/AKT signaling alongside mTORC1 inhibition upon AMPK activation. Mechanistically, activated AMPK phosphorylates FOXO3a at Ser413 and enhances its transcriptional activity, thereby results in upregulation of Rictor, a downstream target and core component of mTORC2. This cascade enhances AKT Ser473 phosphorylation, propagating pro survival and pro proliferative signals (55). Such bidirectional regulation appears seemingly paradoxical given AMPK’s canonical role as an energy sensor that suppresses energy-consuming processes (7). We propose it represents an adaptive compensatory strategy. While mTORC1 inhibition curtails energy consumption by blocking anabolic processes such as protein and lipid biosynthesis, moderate AKT activation via the FOXO3/Rictor/mTORC2 axis preserves essential survival signaling to prevent apoptosis under severe energy depletion. This strategy of “anabolic suppression coupled with preserved survival signaling” may reflect a form of metabolic plasticity evolved to maintain cellular homeostasis. Although comparable bidirectional regulation of mTOR complexes by AMPK has been described previously (56), to the best of our knowledge, this is the first study to link this phenomenon to drug-induced S-phase arrest in ESCC. This discrepancy is primarily attributed.

The differential magnitudes of AKT activation between normal and ESCC cells further explains their distinct cell cycle outcomes. Mechanistically, complete AKT activation requires phosphorylation at both Thr308 and Ser473 (34). In HEEC cells, only Ser473 phosphorylation increased upon AICAR treatment, while Thr308 phosphorylation remained largely unchanged. In contrast, both residues were concurrently phosphorylated in TE1 and TE13 cells. This discrepancy may be attributable, at least in part, to the differential regulation of PTEN, a critical negative regulator of PI3K/AKT signaling that suppresses PDK1-mediated AKT Thr308 phosphorylation by catalyzing PIP3 dephosphorylation (57). In HEEC cells, AICAR treatment upregulated PTEN expression, thereby effectively restraining Thr308 phosphorylation and maintained AKT in a partially activated state. In TE1 and TE13 cells, PTEN levels remained unchanged, allowing full AKT activation. The differential PTEN regulation likely governed by p53 functional status, as PTEN is a direct transcriptional target of p53 (58). The p53-PTEN axis functions as a positive feedback loop wherein p53 binds to the PTEN promoter to drive transcription, while PTEN reciprocally enhances p53 protein stability (59). Consequently, in p53-proficient HEEC cells, AICAR treatment increased p53 and PTEN levels, establishing a protective negative feedback loop. In p53-deficient ESCC cells, the disruption of this feedback loop results in unchecked AKT activation and compromised G1/S checkpoint.

Divergent cell cycle responses induced by AICAR in esophageal epithelial cells and ESCC cells underscores its potential advantages as an antitumor agent. Irinotecan, a topoisomerase I inhibitor, is used as a second-line therapeutic option for ESCC and exerts its cytotoxic effects primarily on S phase cells. However, its clinical utility is often limited by adverse events such as myelosuppression and hepatotoxicity (60). Our results demonstrate that AICAR pretreatment synchronized ESCC cells in the S phase with negligible impacts on the cell cycle distribution of HEEC. These findings suggest that subsequent irinotecan administration might preferentially eliminate malignant cells while sparing normal tissues. This combinatorial strategy may enhance therapeutic efficacy while mitigating off-target toxicity. Similarly, AICAR pretreatment also sensitized ESCC cells to 5-Fu, another S-phase-targeted chemotherapeutic, reducing the effective dose required for comparable cytotoxicity.

Several limitations of the present study warrant acknowledgement. First, although AICAR is widely used as a pharmacological AMPK activator, it also mediates exhibits AMPK-independent off target effects. Further studies employing AMPK knockout or knockdown systems are necessary to verify whether the observed cellular phenotypes are fully reliant on the AMPK signaling. Second, the causal relationship between p53 functional status and differential expression of PTEN, p27^Kip1^ and p21^Cip1^ requires further validation through gain- and loss-of function assays. Specifically, this should involve the restoration of wild-type p53 reconstitution in p53-deficient cells and p53 knockout in normal cells. Third, inference of the AMPK/FOXO3/RICTOR/mTORC2/AKT axis in this work is primarily based on Western blot analysis of protein phosphorylation. Genetic manipulations such as knockdown or overexpression of FOXO3 and Rictor will be required in future investigations to functionally validate this signaling cascade and confirm the causal relationship within this pathway. Finally, *in vivo* animal experiments are needed to optimize dosing regimens and evaluate the antitumor efficacy and safety of AICAR in combination with S-phase-targeted chemotherapeutics such as irinotecan or 5- Fu.

## 5 Conclusion

In summary, this study demonstrates that the AMPK activator AICAR elicits divergent cell cycle effects in esophageal epithelial cells and ESCC cells by concomitantly modulating mTORC1 and mTORC2/AKT signaling. Functional p53 and inducible PTEN constrain AKT activation and prevent S-phase arrest in normal cells, while the loss of these safeguards in ESCC cells leads to unrestrained AKT activation and subsequent S-phase trapping. This distinction difference renders AICAR a promising adjuvant to potentiate S-phase-targeted chemotherapy for ESCC.

## Data Availability

The raw data supporting the conclusions of this article will be made available by the authors, without undue reservation.

## Supplementary material

Supplementary Figure 1 Dose-dependent effects of AICAR on cell viability. Cell viability was measured by the CCK-8 assay after treatment with AICAR at the indicated concentrations (0, 250, 500, and 1000 μM) for 48 h. The 1000 μM concentration caused severe cell death in TE13 cells. Although 250 μM AICAR induced statistically significant but modest antiproliferative effects (less than 20% growth inhibition), this small effect size provides a narrow dynamic window for mechanistic characterization. The 500 μM dose was chosen as the working concentration to achieve robust AMPK activation while retaining sufficient viable cells for subsequent mechanistic experiments. The viability of control cells at 0 μM was set to 100%. Data are presented as mean ± SEM (n = 3).

Supplementary Figure 2 AICAR pretreatment sensitizes ESCC cell lines TE1 and TE13 to 5-Fu. TE1 and TE13 were pretreated with AICAR for 48 hours, followed by drug withdrawal and subsequent treatment with 5-Fu (1 μg/ml) for 48 hours. Apoptosis was assessed by Annexin V/PI double staining. The apoptotic rate in the AICAR pretreatment followed by 5-Fu (1 μg/ml) group was comparable to that in the 5-Fu monotherapy group (5 μg/ml), with no statistically significant difference between the two groups. Data are presented as mean ± SD (n = 3). ns, not significant.

## References

[1] Jin W Huang K Ding Z Zhang M Li C Yuan Z . Global, regional, and national burden of esophageal cancer: a systematic analysis of the Global Burden of Disease Study 2021. biomark Res. (2025) 13:3. doi: 10.1186/s40364-024-00718-2 39762900 PMC11702276

[2] Waters J Kreznik S Reznik I . Update on management of squamous cell esophageal cancer. Curr Oncol Rep. (2022) 24:375–85. doi: 10.1007/s11912-021-01153-4 35142974

[3] Sheikh M Roshandel G McCormack V Malekzadeh R . Current status and future prospects for esophageal cancer. Cancers (Basel). (2023) 15:765. doi: 10.3390/cancers15030765 36765722 PMC9913274

[4] van Hagen P Hulshof MC van Lanschot JJ Steyerberg EW van Berge Henegouwen MI Wijnhoven BP . Preoperative chemoradiotherapy for esophageal or junctional cancer. N Engl J Med. (2012) 366:2074–84. doi: 10.1056/NEJMoa1112088 22646630

[5] Hardie DG Carling D . The AMP-activated protein kinase--fuel gauge of the mammalian cell? Eur J Biochem. (1997) 246:259–73. doi: 10.1111/j.1432-1033.1997.00259.x 9208914

[6] Kemp BE Stapleton D Campbell DJ Chen ZP Murthy S Walter M . AMP-activated protein kinase, super metabolic regulator. Biochem Soc Trans. (2003) 31:162–8. doi: 10.1042/bst0310162 12546677

[7] Hardie DG Ross FA Hawley SA . AMPK: a nutrient and energy sensor that maintains energy homeostasis. Nat Rev Mol Cell Biol. (2012) 13:251–62. doi: 10.1038/nrm3311 22436748 PMC5726489

[8] Inoki K Zhu T Guan KL . TSC2 mediates cellular energy response to control cell growth and survival. Cell. (2003) 115:577–90. doi: 10.1016/s0092-8674(03)00929-2 14651849

[9] Greer EL Oskoui PR Banko MR Maniar JM Gygi MP Gygi SP . The energy sensor AMP-activated protein kinase directly regulates the mammalian FOXO3 transcription factor. J Biol Chem. (2007) 282:30107–19. doi: 10.1074/jbc.M705325200 17711846

[10] Villanueva-Paz M Cotan D Garrido-Maraver J Oropesa-Avila M de la Mata M Delgado-Pavon A . AMPK regulation of cell growth, apoptosis, autophagy, and bioenergetics. Exp Suppl. (2016) 107:45–71. doi: 10.1007/978-3-319-43589-3_3 27812976

[11] Zhang P Song Y Sun Y Li X Chen L Yang L . AMPK/GSK3beta/beta-catenin cascade-triggered overexpression of CEMIP promotes migration and invasion in anoikis-resistant prostate cancer cells by enhancing metabolic reprogramming. FASEB J. (2018) 32:3924–35. doi: 10.1096/fj.201701078R 29505302

[12] Rehman G Shehzad A Khan AL Hamayun M . Role of AMP-activated protein kinase in cancer therapy. Arch Pharm (Weinheim). (2014) 347:457–68. doi: 10.1002/ardp.201300402 24677093

[13] Fogarty S Hardie DG . Development of protein kinase activators: AMPK as a target in metabolic disorders and cancer. Biochim Biophys Acta. (2010) 1804:581–91. doi: 10.1016/j.bbapap.2009.09.012 19778642

[14] Mohanty SS Warrier S Rangarajan A . Rethinking AMPK: A reversible switch fortifying cancer cell stress-resilience. Yale J Biol Med. (2025) 98:33–52. doi: 10.59249/JKBB6336 40165808 PMC11952127

[15] Hsu CC Peng D Cai Z Lin HK . AMPK signaling and its targeting in cancer progression and treatment. Semin Cancer Biol. (2022) 85:52–68. doi: 10.1016/j.semcancer.2021.04.006 33862221 PMC9768867

[16] Sullivan JE Brocklehurst KJ Marley AE Carey F Carling D Beri RK . Inhibition of lipolysis and lipogenesis in isolated rat adipocytes with AICAR, a cell-permeable activator of AMP-activated protein kinase. FEBS Lett. (1994) 353:33–6. doi: 10.1016/0014-5793(94)01006-4 7926017

[17] Visnjic D Lalic H Dembitz V Tomic B Smoljo T . AICAr, a widely used AMPK activator with important AMPK-independent effects: A systematic review. Cells. (2021) 10:1095. doi: 10.3390/cells10051095 34064363 PMC8147799

[18] Jose C Hebert-Chatelain E Bellance N Larendra A Su M Nouette-Gaulain K . AICAR inhibits cancer cell growth and triggers cell-type distinct effects on OXPHOS biogenesis, oxidative stress and Akt activation. Biochim Biophys Acta. (2011) 1807:707–18. doi: 10.1016/j.bbabio.2010.12.002 21692240

[19] Guan TJ Qin FJ Du JH Geng L Zhang YY Li M . AICAR inhibits proliferation and induced S-phase arrest, and promotes apoptosis in CaSki cells. Acta Pharmacol Sin. (2007) 28:1984–90. doi: 10.1111/j.1745-7254.2007.00675.x 18031613

[20] Theodoropoulou S Brodowska K Kayama M Morizane Y Miller JW Gragoudas ES . Aminoimidazole carboxamide ribonucleotide (AICAR) inhibits the growth of retinoblastoma *in vivo* by decreasing angiogenesis and inducing apoptosis. PloS One. (2013) 8:e52852. doi: 10.1371/journal.pone.0052852 23300996 PMC3536763

[21] Sauer H Engel S Milosevic N Sharifpanah F Wartenberg M . Activation of AMP-kinase by AICAR induces apoptosis of DU-145 prostate cancer cells through generation of reactive oxygen species and activation of c-Jun N-terminal kinase. Int J Oncol. (2012) 40:501–8. doi: 10.3892/ijo.2011.1230 22002081

[22] Persons KS Eddy VJ Chadid S Deoliveira R Saha AK Ray R . Anti-growth effect of 1,25-dihydroxyvitamin D3-3-bromoacetate alone or in combination with 5-amino-imidazole-4-carboxamide-1-beta-4-ribofuranoside in pancreatic cancer cells. Anticancer Res. (2010) 30:1875–80. 20651329

[23] Bardeleben C Sharma S Reeve JR Bassilian S Frost P Hoang B . Metabolomics identifies pyrimidine starvation as the mechanism of 5-aminoimidazole-4-carboxamide-1-beta-riboside-induced apoptosis in multiple myeloma cells. Mol Cancer Ther. (2013) 12:1310–21. doi: 10.1158/1535-7163.MCT-12-1042 23585020 PMC3707969

[24] Tripathi V Jaiswal P Assaiya A Kumar J Parmar HS . Anti-cancer effects of 5-aminoimidazole-4-carboxamide-1-beta-d-ribofuranoside (AICAR) on triple-negative breast cancer (TNBC) cells: Mitochondrial modulation as an underlying mechanism. Curr Cancer Drug Targets. (2022) 22:245–56. doi: 10.2174/1568009622666220207101212 35135451

[25] Woodard J Platanias LC . AMP-activated kinase (AMPK)-generated signals in malignant melanoma cell growth and survival. Biochem Biophys Res Commun. (2010) 398:135–9. doi: 10.1016/j.bbrc.2010.06.052 20599746 PMC2927205

[26] Xiang X Saha AK Wen R Ruderman NB Luo Z . AMP-activated protein kinase activators can inhibit the growth of prostate cancer cells by multiple mechanisms. Biochem Biophys Res Commun. (2004) 321:161–7. doi: 10.1016/j.bbrc.2004.06.133 15358229

[27] Sengupta TK Leclerc GM Hsieh-Kinser TT Leclerc GJ Singh I Barredo JC . Cytotoxic effect of 5-aminoimidazole-4-carboxamide-1-beta-4-ribofuranoside (AICAR) on childhood acute lymphoblastic leukemia (ALL) cells: implication for targeted therapy. Mol Cancer. (2007) 6:46. doi: 10.1186/1476-4598-6-46 17623090 PMC1948012

[28] Yu SY Chan DW Liu VW Ngan HY . Inhibition of cervical cancer cell growth through activation of upstream kinases of AMP-activated protein kinase. Tumour Biol. (2009) 30:80–5. doi: 10.1159/000216843 19407487

[29] Mukherjee P Mulrooney TJ Marsh J Blair D Chiles TC Seyfried TN . Differential effects of energy stress on AMPK phosphorylation and apoptosis in experimental brain tumor and normal brain. Mol Cancer. (2008) 7:37. doi: 10.1186/1476-4598-7-37 18474106 PMC2397440

[30] Su CC Hsieh KL Liu PL Yeh HC Huang SP Fang SH . AICAR induces apoptosis and inhibits migration and invasion in prostate cancer cells through an AMPK/mTOR-dependent pathway. Int J Mol Sci. (2019) 20:1647. doi: 10.3390/ijms20071647 30987073 PMC6480054

[31] Lin A Piao HL Zhuang L Sarbassov dos D Ma L Gan B . FoxO transcription factors promote AKT Ser473 phosphorylation and renal tumor growth in response to pharmacologic inhibition of the PI3K-AKT pathway. Cancer Res. (2014) 74:1682–93. doi: 10.1158/0008-5472.CAN-13-1729 24448243 PMC4087030

[32] Kim LC Rhee CH Chen J . RICTOR amplification promotes NSCLC cell proliferation through formation and activation of mTORC2 at the expense of mTORC1. Mol Cancer Res. (2020) 18:1675–84. doi: 10.1158/1541-7786.MCR-20-0262 32801163 PMC7642103

[33] Alessi DR Andjelkovic M Caudwell B Cron P Morrice N Cohen P . Mechanism of activation of protein kinase B by insulin and IGF-1. EMBO J. (1996) 15:6541–51. doi: 10.1002/j.1460-2075.1996.tb01045.x 8978681 PMC452479

[34] Bellacosa A Chan TO Ahmed NN Datta K Malstrom S Stokoe D . Akt activation by growth factors is a multiple-step process: the role of the PH domain. Oncogene. (1998) 17:313–25. doi: 10.1038/sj.onc.1201947 9690513

[35] Manning BD Cantley LC . AKT/PKB signaling: navigating downstream. Cell. (2007) 129:1261–74. doi: 10.1016/j.cell.2007.06.009 17604717 PMC2756685

[36] Ono H Katagiri H Funaki M Anai M Inukai K Fukushima Y . Regulation of phosphoinositide metabolism, Akt phosphorylation, and glucose transport by PTEN (phosphatase and tensin homolog deleted on chromosome 10) in 3T3-L1 adipocytes. Mol Endocrinol. (2001) 15:1411–22. doi: 10.1210/mend.15.8.0684 11463863

[37] Magnuson B Ekim B Fingar DC . Regulation and function of ribosomal protein S6 kinase (S6K) within mTOR signalling networks. Biochem J. (2012) 441:1–21. doi: 10.1042/BJ20110892 22168436

[38] Burnett PE Barrow RK Cohen NA Snyder SH Sabatini DM . RAFT1 phosphorylation of the translational regulators p70 S6 kinase and 4E-BP1. Proc Natl Acad Sci USA. (1998) 95:1432–7. doi: 10.1073/pnas.95.4.1432 9465032 PMC19032

[39] Gonzalez A Hall MN Lin SC Hardie DG . AMPK and TOR: The Yin and Yang of cellular nutrient sensing and growth control. Cell Metab. (2020) 31:472–92. doi: 10.1016/j.cmet.2020.01.015 32130880

[40] Motoshima H Goldstein BJ Igata M Araki E . AMPK and cell proliferation--AMPK as a therapeutic target for atherosclerosis and cancer. J Physiol. (2006) 574:63–71. doi: 10.1113/jphysiol.2006.108324 16613876 PMC1817805

[41] Zhuang Y Miskimins WK . Cell cycle arrest in Metformin treated breast cancer cells involves activation of AMPK, downregulation of cyclin D1, and requires p27Kip1 or p21Cip1. J Mol Signal. (2008) 3:18. doi: 10.1186/1750-2187-3-18 19046439 PMC2613390

[42] Cho AR Park WY Lee HJ Sim DY Im E Park JE . Antitumor effect of Morusin via G1 arrest and antiglycolysis by AMPK activation in hepatocellular cancer. Int J Mol Sci. (2021) 22:10619. doi: 10.3390/ijms221910619 34638959 PMC8508967

[43] Isakovic A Harhaji L Stevanovic D Markovic Z Sumarac-Dumanovic M Starcevic V . Dual antiglioma action of metformin: cell cycle arrest and mitochondria-dependent apoptosis. Cell Mol Life Sci. (2007) 64:1290–302. doi: 10.1007/s00018-007-7080-4 17447005 PMC11136022

[44] Liu J Li M Song B Jia C Zhang L Bai X . Metformin inhibits renal cell carcinoma *in vitro* and *in vivo* xenograft. Urol Oncol. (2013) 31:264–70. doi: 10.1016/j.urolonc.2011.01.003 21676631

[45] Jones RG Plas DR Kubek S Buzzai M Mu J Xu Y . AMP-activated protein kinase induces a p53-dependent metabolic checkpoint. Mol Cell. (2005) 18:283–93. doi: 10.1016/j.molcel.2005.03.027 15866171

[46] Kim YC Day RM . Angiotensin II regulates activation of Bim via Rb/E2F1 during apoptosis: involvement of interaction between AMPKbeta1/2 and Cdk4. Am J Physiol Lung Cell Mol Physiol. (2012) 303:L228–38. doi: 10.1152/ajplung.00087.2012 22659879 PMC3423861

[47] Gwinn DM Shackelford DB Egan DF Mihaylova MM Mery A Vasquez DS . AMPK phosphorylation of raptor mediates a metabolic checkpoint. Mol Cell. (2008) 30:214–26. doi: 10.1016/j.molcel.2008.03.003 18439900 PMC2674027

[48] Medema RH Kops GJ Bos JL Burgering BM . AFX-like Forkhead transcription factors mediate cell-cycle regulation by Ras and PKB through p27kip1. Nature. (2000) 404:782–7. doi: 10.1038/35008115 10783894

[49] Hauck L Harms C Grothe D An J Gertz K Kronenberg G . Critical role for FoxO3a-dependent regulation of p21CIP1/WAF1 in response to statin signaling in cardiac myocytes. Circ Res. (2007) 100:50–60. doi: 10.1161/01.RES.0000254704.92532.b9 17158337

[50] Schmidt M Fernandez de Mattos S van der Horst A Klompmaker R Kops GJ Lam EW . Cell cycle inhibition by FoxO forkhead transcription factors involves downregulation of cyclin D. Mol Cell Biol. (2002) 22:7842–52. doi: 10.1128/MCB.22.22.7842-7852.2002 12391153 PMC134724

[51] Renault VM Thekkat PU Hoang KL White JL Brady CA Kenzelmann Broz D . The pro-longevity gene FoxO3 is a direct target of the p53 tumor suppressor. Oncogene. (2011) 30:3207–21. doi: 10.1038/onc.2011.35 21423206 PMC3136551

[52] Miyaguchi Y Tsuchiya K Sakamoto K . P53 negatively regulates the transcriptional activity of FOXO3a under oxidative stress. Cell Biol Int. (2009) 33:853–60. doi: 10.1016/j.cellbi.2009.04.017 19427386

[53] Barnas C Martel-Planche G Furukawa Y Hollstein M Montesano R Hainaut P . Inactivation of the p53 protein in cell lines derived from human esophageal cancers. Int J Cancer. (1997) 71:79–87. doi: 10.1002/(sici)1097-0215(19970328)71:1<79::aid-ijc14>3.0.co;2-4 9096669

[54] Cargnello M Tcherkezian J Roux PP . The expanding role of mTOR in cancer cell growth and proliferation. Mutagenesis. (2015) 30:169–76. doi: 10.1093/mutage/geu045 25688110 PMC5943824

[55] Ragupathi A Kim C Jacinto E . The mTORC2 signaling network: targets and cross-talks. Biochem J. (2024) 481:45–91. doi: 10.1042/BCJ20220325 38270460 PMC10903481

[56] Sadria M Seo D Layton AT . The mixed blessing of AMPK signaling in Cancer treatments. BMC Cancer. (2022) 22:105. doi: 10.1186/s12885-022-09211-1 35078427 PMC8786626

[57] Georgescu MM . PTEN tumor suppressor network in PI3K-akt pathway control. Genes Cancer. (2010) 1:1170–7. doi: 10.1177/1947601911407325 21779440 PMC3092286

[58] Stambolic V MacPherson D Sas D Lin Y Snow B Jang Y . Regulation of PTEN transcription by p53. Mol Cell. (2001) 8:317–25. doi: 10.1016/s1097-2765(01)00323-9 11545734

[59] Mayo LD Dixon JE Durden DL Tonks NK Donner DB . PTEN protects p53 from Mdm2 and sensitizes cancer cells to chemotherapy. J Biol Chem. (2002) 277:5484–9. doi: 10.1074/jbc.M108302200 11729185

[60] Pommier Y . DNA topoisomerase I inhibitors: chemistry, biology, and interfacial inhibition. Chem Rev. (2009) 109:2894–902. doi: 10.1021/cr900097c 19476377 PMC2707511

